# Mapping the Brazilian scientific output in oncology: a bibliometric study on cancer research

**DOI:** 10.1590/1806-9282.20250669

**Published:** 2026-01-09

**Authors:** Layza Hellen Fernandes Menezes, Italo Carvalho Martins, Welbert Souza Furtado, Breno Dias Lima Ribeiro, Maria Júlia de Sena Lopes, Consuelo Penha Castro Marques, Caio Márcio Barros de Oliveira, Plínio da Cunha Leal

**Affiliations:** 1 Center for Sciences of Pinheiro – Pinheiro (MA), Brazil.; 2 Universidade Federal do Maranhão, Department of Medicine – São Luís (MA), Brazil.

## INTRODUCTION

Cancer stands as one of the leading causes of mortality worldwide and is recognized as a major public health issue. In Brazil, it is estimated that more than 700,000 new cancer cases will be diagnosed between 2023 and 2025^1^. In this context, scientific knowledge becomes an essential tool for advancing therapies and formulating more effective public health policies.

The understanding of cancer in Brazil has undergone several transformations over time. At certain points, it was regarded as a disease limited to the elite, and later, during the 20th century, it was even perceived as a contagious illness. Nonetheless, national medical journals such as Brasil Médico, Revista Médica de São Paulo, and Gazeta Médica da Bahia played a crucial role in disseminating the scientific knowledge of the time, contributing to improvements in cancer care despite structural limitations. As scientific research progressed, it became evident that cancer epidemiology was more closely related to lifestyle factors and heredity. However, until this understanding was firmly established in the literature, many Brazilians relied solely on traditional medicine^2,3^.

The creation of the National Cancer Institute (INCA) marked a milestone in the country's approach to cancer, expanding its role beyond assistance to include the promotion of oncological research, especially from the 1980s onward, when the institution was restructured and supported by public investment^4^. From that point, various governmental initiatives were implemented to produce, organize, and disseminate cancer-related information in Brazil^5^. Furthermore, the development of data repositories enabled significant progress in patient care^6^.

Scientific development is vital for expanding clinical knowledge, particularly in the health sciences. Evidence-based medicine not only improves patient outcomes but also provides greater confidence and safety for healthcare professionals in their practice. Given that oncology has long intrigued researchers—due to both the diversity of its manifestations and the lack of a definitive cure—research remains a fundamental tool to navigate this complex and evolving field.

Therefore, understanding the landscape of Brazilian scientific production in oncology is essential to identify research gaps and trends. Bibliometric analysis, widely recognized as an effective method for quantifying and evaluating scientific output across various disciplines, allows for the mapping of research development and its comparison across different contexts. In light of this, we aimed to examine studies with Brazilian contributions through a bibliometric approach, in order to assess the real impact of national publications in international journals.

## METHODS

This study is a bibliometric analysis based on data collected from the Scopus database (Elsevier) in October 2024. The choice of Scopus was motivated by its advanced filtering capabilities, particularly through the "Sources" tab, which allows journals to be selected by specific subject areas. For this study, the subject area "Oncology" was chosen, and the search was further refined by applying the country filter for Brazil. Among the journals retrieved, only those with the highest CiteScore were selected, ensuring the inclusion of publications from high-impact and internationally recognized sources in the oncology field.

The final selection included journals such as CA: A Cancer Journal for Clinicians, Nature Reviews Cancer, Nature Reviews Clinical Oncology, Annals of Oncology, and The Lancet Oncology. The search was restricted to the period from 1990 to 2023, and errata and conference abstracts were excluded. All types of study designs were included to provide a comprehensive overview of the scientific output.

The bibliometric metadata were exported in BibTeX format and initially organized in Microsoft Excel (version 2023) for data cleaning and structuring. The subsequent analysis was performed using R Studio (version 4.2.3, R Foundation for Statistical Computing, Vienna, Austria) with the bibliometrix package, which enabled the quantification of publication metrics and the generation of descriptive statistics. For the construction of co-authorship networks and thematic maps, VOSviewer software was employed. Visual representations such as graphs and charts were created using Google Sheets.

The topics analyzed were: the total number of publications (1990–2023), state of publication (based on the first author's state), interstate contributions, international collaboration, publication topics, study design, article accessibility, and the contribution of Brazilian authors.

Co-authorship relationships were analyzed based on the number of co-authored documents, evaluating collaboration between researchers from different countries and among authors who published together. Only countries with at least five publications and authors with at least four collaborative articles were considered. The proximity of authors in the graphs reflects higher collaboration frequency.

Co-occurrence analysis was determined by the number of documents in which two topics appeared together, assessing the frequency of their simultaneous citation in other works. Citation analysis included only authors cited at least 20 times, with proximity in the graphs indicating a higher frequency of joint citations. An analysis was also conducted to measure the citation degree between countries, considering those with at least five published articles.

Bibliographic coupling analysis was based on the number of shared references between articles. Co-citation relationships were evaluated based on the number of times two authors were cited together, reflecting thematic proximity between them.

This set of analyses aims to understand patterns of scientific collaboration, the impact of Brazilian publications, and citation dynamics within the field of oncology.

## RESULTS

**Figure 1:**
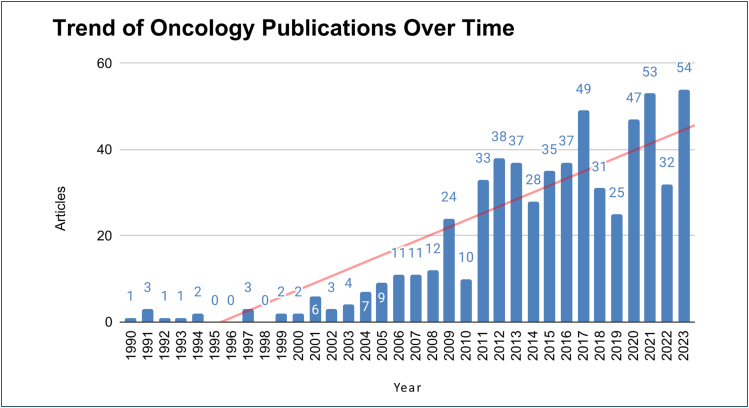
Annual scientific production. Graph showing the number of oncology articles published by Brazilian researchers in major scientific journals (1990–2024). The trendline represents the growth of scientific production over the years.

It is possible to observe a nonlinear growth in the number of published articles since 1990. Notably, from 2009 onward, there was a trend that exceeded the expected publication rate for that year. After this period, successive increases and decreases in the number of articles can be observed, which may indicate that the writing and publication process takes longer than a year to be completed (Figure 1).

**Figure 2:**
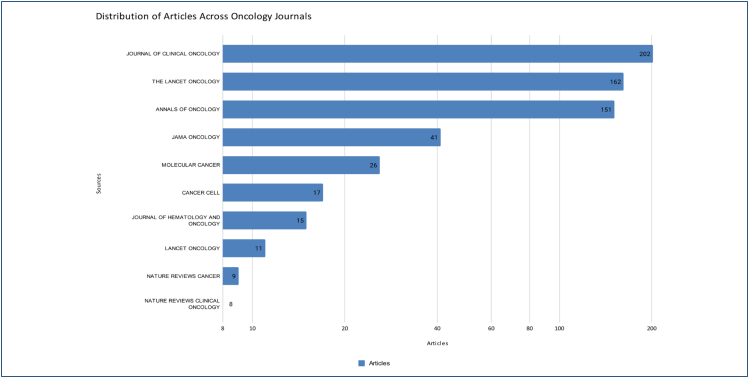
Most relevant sources. Graph showing the number of oncology articles published by Brazilian researchers in high-impact journals. The values represent the total count of publications per journal.

**Figure 3:**
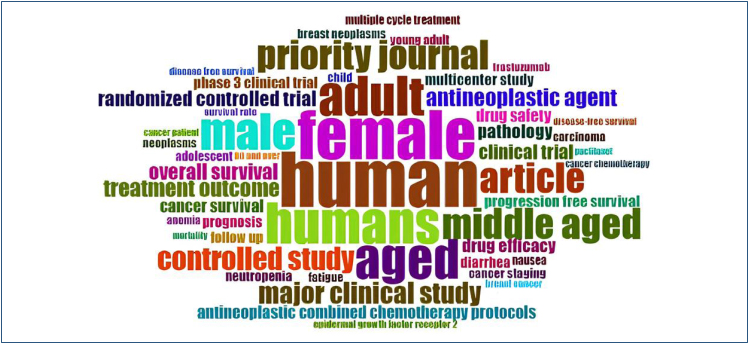
WordCloud. Word cloud representing the main terms associated with oncology articles published by Brazilian researchers. The word sizes reflect the frequency of occurrence of the terms in the analyzed studies.

The concentration of article publications among three sources is notable: Journal of Clinical Oncology with 202 articles, The Lancet Oncology with 162 articles, and Annals of Oncology with 151 articles. Together, these three sources account for 493 articles, 100 publications more than the sum of all the others combined, highlighting the greater prominence of these journals. Additionally, JAMA Oncology contributes 41 articles, Molecular Cancer 26, Cancer Cell 17, and Journal of Hematology and Oncology 15. With a more modest participation, Lancet On has 11 articles, while Nature Re and Nature Re have 8 and 9, respectively. All these sources originate from the United States or the United Kingdom, reflecting a well-established scenario in the current literature regarding the relevance of publications in the English language (Figure 2). The most recurring terms found in the publications include "cancer," "immunotherapy," "treatment," and "biomarkers," which are visually represented in the word cloud in Figure 3. This indicates that these are the main focuses of oncological research in Brazil

**Figure 4:**
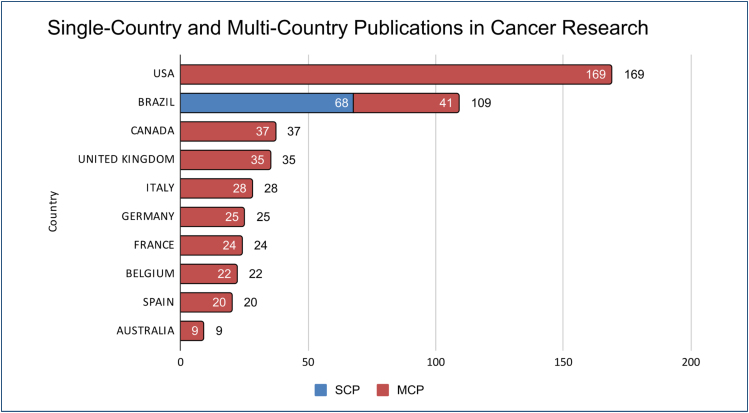
Multiple-country publications (MCPs) and single-country publications (SCPs). Distribution of the number of oncology articles by country, distinguishing between single-country publications (SCPs) and multiple-country publications (MCPs). The values represent the total publication count per country.

Figure 4 illustrates the distribution of scientific publications in cancer research, differentiating between articles authored by researchers from a single country (single-country publications [SCPs]) and those resulting from multi-country collaborations (multiple-country publications [MCPs]). The horizontal axis represents the number of articles, while the vertical axis lists the contributing countries. The data reveal that the United States leads in MCPs, with 169 MCP articles. Brazil shows a significant contribution, with 68 SCPs and 41 MCPs, totaling 109 articles. Other prominent countries in multi-country collaborations include Canada (37 MCP articles), the United Kingdom (35 MCP articles), Italy (28 MCP articles), Germany (25 MCP articles), France (24 MCP articles), Belgium (22 MCP articles), Spain (20 MCP articles), and Australia (9 MCP articles). This visualization underscores the highly collaborative nature of global cancer research, highlighting Brazil's notable engagement in both national and international scientific endeavors.

**Figure 5:**
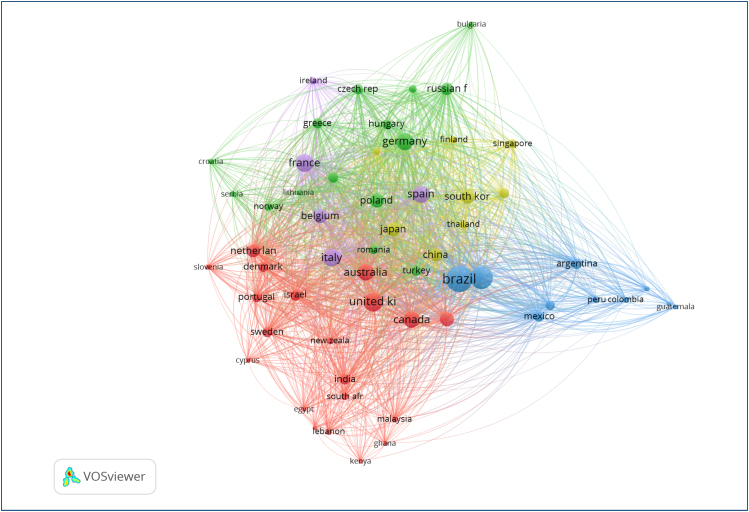
International collaboration map in oncology by country (minimum five publications).

Figure 5 illustrates the country co-authorship network in oncology research, including only nations with a minimum of five published documents in the analyzed database. The map reveals a complex and interconnected international collaboration structure, where several distinct clusters emerged, reflecting predominant partnership patterns. Notably, a blue cluster predominantly groups Latin American countries (e.g., Argentina, Colombia, and Mexico), while vast red and purple clusters encompass major research centers from Europe, North America, and Asia, such as the United Kingdom, Germany, France, China, Japan, and Canada, characterized by a dense network of collaborations. Brazil (a prominent node) occupies a central and strategic position, demonstrating strong co-authorship ties with both its South American neighbors within the blue cluster and with global scientific powers present in the European and Asian clusters, such as Portugal and China. The magnitude and diversity of these connections highlight Brazil's active role and the broad integration of its partnerships in global oncology research.

## DISCUSSION

The results of this analysis highlight a strong collaborative profile in scientific publications on oncology with Brazilian participation. As depicted in Figure 4, while Brazil maintains significant SCPs, its engagement in multi-country collaborations is also substantial. Collaboration with the United States stands out as the most significant partner, reflecting its established global leadership in scientific research, largely supported by substantial public and private investments^7,8^. Additionally, European countries, particularly from the Mediterranean region, such as Italy, France, and Spain, also emerge as relevant partners, demonstrating the integration of Brazilian science into global networks of excellence. Strengthening partnerships among Latin American countries, as a form of South-South cooperation, could further boost the region's scientific production and promote multicenter studies tailored to local demands. Beyond fostering regional cooperation, this strategy holds the potential to enhance the impact of Latin American science on the global stage, increasing its relevance and contributing to innovative solutions that are context-specific and globally valuable^9^.

On the other hand, the concentration of publications in high-impact journals, such as Journal of Clinical Oncology, The Lancet Oncology, and Annals of Oncology, demonstrates a consistent effort to expand the visibility of Brazilian research. However, the notable absence of journals from developing countries, including Brazil and China, among these leading publication outlets is a significant finding. This phenomenon aligns with observations in other specialized fields, such as the scientific production on follicular thyroid carcinoma, as suggested by Fan et al^10^. The predominance of publications in a select group of highly influential journals suggests a strategic focus on well-established channels, which inherently favors global visibility and citation impact. Brazilian researchers have likely adopted this publication strategy to maximize the reach of their studies and ensure their findings are disseminated through highly prestigious platforms, given that international exposure and citation counts are directly correlated with the choice of journals possessing a high impact factor^11^. This reliance on external platforms, however, also highlights the persistent challenges faced by journals from the Global South in achieving similar international recognition and impact.

The number of publications showed a modest increase between 1990 and 2008; however, from 2009 onward, there was a more pronounced, though unstable, growth in oncology-related scientific production. In most years during this period, over 24 studies were published annually, indicating that the topic has gained greater attention and has been more widely studied^11^.

The most recurring terms in publications include "cancer," "immunotherapy," "treatment," "chemotherapy," and "biomarkers." The field of immunotherapy has been extensively studied and has shown promising results; however, despite the relevance of the research conducted so far, gaps remain, such as a detailed understanding of the relationship between different immunotherapies and the reduction of tumor burden^12^. Additionally, terms such as "PD-1," "targeted therapy," and "breast cancer" stand out, as they represent specific research areas with a high volume of studies, establishing themselves as promising fields within oncology^13^. Regarding programmed cell death protein 1 (PD-1), for instance, major discussions revolve around the efficacy and prognosis of various tumors after treatment with PD-1 and programmed death-ligand 1 (PD-L1) inhibitors, as well as the activation and expression of essential factors in the anti-tumor immune process^14^.

This study has some limitations. First, the research was based exclusively on data from the Scopus platform, which may have reduced the scope of the results. The use of other databases such as Web of Science or PubMed/Medline could have provided a more in-depth and comprehensive analysis. Additionally, the temporal restriction of the data in this bibliometric analysis may have led to the exclusion of more recent studies, potentially impacting the representativeness of the findings.

## CONCLUSION

Brazil is making a significant and expanding contribution to international oncology research, primarily through strategic collaborations with leading countries like the United States and European nations. These partnerships are essential for national scientific advancement, offering access to advanced technologies and enhanced global visibility. Nevertheless, the study highlights two key areas for improvement: a limited focus on local public health challenges, such as high-prevalence cancers within Brazil, and the notable underrepresentation of Brazilian journals among the top international publication outlets. This suggests a strategic reliance on international channels to maximize global reach. Moving forward, it is crucial to balance international collaborations with a reinforced commitment to strengthening national publications and conducting research more aligned with Brazil's unique epidemiological context and treatment needs. This dual approach—integrating global efforts with a strong focus on local demands—will further consolidate Brazil's impactful role in the global oncology landscape.

## Data Availability

The datasets generated and/or analyzed during the current study are available from the corresponding author upon reasonable request.
